# Root and Rhizosphere Bacterial Phosphatase Activity Varies with Tree Species and Soil Phosphorus Availability in Puerto Rico Tropical Forest

**DOI:** 10.3389/fpls.2017.01834

**Published:** 2017-10-30

**Authors:** Kristine G. Cabugao, Collin M. Timm, Alyssa A. Carrell, Joanne Childs, Tse-Yuan S. Lu, Dale A. Pelletier, David J. Weston, Richard J. Norby

**Affiliations:** ^1^Bredesen Center for Interdisciplinary Research and Graduate Education, University of Tennessee, Knoxville, TN, United States; ^2^Environmental Sciences Division, Oak Ridge National Laboratory, Oak Ridge, TN, United States; ^3^Climate Change Science Institute, Oak Ridge National Laboratory, Oak Ridge, TN, United States; ^4^Biosciences Division, Oak Ridge National Laboratory, Oak Ridge, TN, United States

**Keywords:** nutrient acquisition, phosphatase, phosphorus, plant growth promoting rhizobacteria, plant microbiome, root functional traits

## Abstract

Tropical forests generally occur on highly weathered soils that, in combination with the immobility of phosphorus (P), often result in soils lacking orthophosphate, the form of P most easily metabolized by plants and microbes. In these soils, mineralization of organic P can be the major source for orthophosphate. Both plants and microbes encode for phosphatases capable of mineralizing a range of organic P compounds. However, the activity of these enzymes depends on several edaphic factors including P availability, tree species, and microbial communities. Thus, phosphatase activity in both roots and the root microbial community constitute an important role in P mineralization and P nutrient dynamics that are not well studied in tropical forests. To relate phosphatase activity of roots and bacteria in tropical forests, we measured phosphatase activity in roots and bacterial isolates as well as bacterial community composition from the rhizosphere. Three forests in the Luquillo Mountains of Puerto Rico were selected to represent a range of soil P availability as measured using the resin P method. Within each site, a minimum of three tree species were chosen to sample. Root and bacterial phosphatase activity were both measured using a colorimetric assay with para-nitrophenyl phosphate as a substrate for the phosphomonoesterase enzyme. Both root and bacterial phosphatase were chiefly influenced by tree species. Though tree species was the only significant factor in root phosphatase activity, there was a negative trend between soil P availability and phosphatase activity in linear regressions of average root phosphatase and resin P. Permutational multivariate analysis of variance of bacterial community composition based on 16S amplicon sequencing indicated that bacterial composition was strongly controlled by soil P availability (*p*-value < 0.05). These results indicate that although root and bacterial phosphatase activity were influenced by tree species; bacterial community composition was chiefly influenced by P availability. Although the sample size is limited given the tremendous diversity of tropical forests, our study indicates the importance of roots and bacterial function to understanding phosphatase activity. Future work will broaden the diversity of tree species and microbial members sampled to provide insight into P mineralization and model representation of tropical forests.

## Introduction

Tropical forests annually accumulate the highest amount of biomass among terrestrial ecosystems, constituting a significant global carbon sink. In 2011, tropical forests were estimated to have stored 262.1 Pg C in live above- and belowground biomass. In contrast, boreal forests contained 53.9 Pg C and temperate forests, 46.6 Pg C (Pan et al., 2013). Paradoxically, many tropical forests occur on highly weathered soils with lower amounts of total P and a higher fraction of occluded (and unavailable) P (Yang et al., 2013). Although both nitrogen (N) and P are likely to co-limit natural ecosystems, P is of particular interest because soil P cannot be replenished biologically in the way that N fixation, prevalent in the tropics, can enhance available N (Cleveland et al., 2011; Townsend et al., 2011). Orthophosphate (H_2_PO_4_^-^ and HPO_4_^2-^) is the form of P most readily available for plant and microbial uptake, vital for many compounds involved in energy transfer, metabolism, membrane transport, signaling, and in the formation of nucleic acids (Raven, 2015). However, prolonged warm and moist climate of the tropics encourage high weathering rates, which deplete sources of P. In addition, P forms insoluble complexes with aluminum and iron ions, both of which are considered geochemical sinks for phosphate due to their specific surface area (Chacon et al., 2006). High weathering rates and the strong reaction between soil iron and aluminum to phosphate result in tropical soils typically devoid of orthophosphate (Vitousek and Sanford, 1984; Chacon et al., 2006; Dalling et al., 2016). Therefore, it is critical to understand factors that regulate P availability given that P limitation could modulate important tropical ecosystem properties such as photosynthetic activity (Bloomfield et al., 2008), interactions with the N cycle, belowground C cycling, litter decomposition, soil organic matter turnover (Cleveland et al., 2011), growth rate of young trees and seedling survival (Alvarez-Clare et al., 2013), respiration (White and Hammond, 2008), tree distribution (Condit et al., 2013), and microbial biomass and composition (Liu et al., 2012). The immense diversity and vitality of tree species in tropical forests suggests the existence of multiple mechanisms that can alleviate limitations in soil P supply (Lynch and Brown, 2001; Lambers et al., 2006). Although interactions between roots and microbes are important to regulating P acquisition, the correlation between root and microbial traits and their influence on soil P are not well understood (Marschner et al., 2011; Mommer and Weemstra, 2012; Iversen, 2014).

The plant microbiome plays an active role in shaping root and soil characteristics that modulate plant P acquisition by altering existing pathways and providing new biochemical capabilities (Bulgarelli et al., 2013). The direct pathway for P uptake is through root hairs or the root epidermis, restricting P uptake to the soil volume closest to the root surface. However, both arbuscular mycorrhizal fungi (AMF) and plant growth promoting bacteria influence this process in a variety of ways. While there has been much attention in relating mycorrhizae to root traits with regards to P acquisition (Valverde-Barrantes et al., 2013; Comas et al., 2014; Kong et al., 2014), less attention has focused on bacterial members of the microbial community and the ways in which they also shape the root system. Many members in the plant growth promoting bacteria group can alter root architecture and morphology by synthesizing major plant hormones such as auxin and ethylene, stimulating the formation of lateral roots, decreasing primary root length, and increasing root hairs – indirectly influencing the effectiveness of soil foraging (Bulgarelli et al., 2013; Niu et al., 2013; Vacheron et al., 2013). In addition, bacteria also directly enhance P availability in the immediate vicinity of the root. P occurs in both inorganic and organic forms. Orthophosphate availability from minerals (inorganic) is highly dependent on soil pH and by soil processes such as sorption–desorption and dissolution–precipitation. Bacteria are capable of solubilizing P from these minerals to enhance P availability in the rhizosphere directly. Bacterial release of protons and secondary metabolites such as organic acid anions and amino acids solubilize P from minerals, influencing sorption–desorption and dissolution–precipitation equilibria in the soil (Bunemann et al., 2004; Vacheron et al., 2013). However, in tropical regions, mineralization of organic P is the major source for orthophosphate (Vitousek and Sanford, 1984).

Plants and microbes release phosphatase enzymes to mineralize organic P compounds. Phosphatase activity refers to the actions of two complementary, but distinct enzymes: phosphodiesterase (PDE) and phosphomonoesterase (PME). PDE hydrolyses complex organic P compounds such as nucleic acids and phospholipids into phosphomonoesters (mononucleotides and inositol phosphates). PME further mineralizes these compounds into orthophosphate which can be directly absorbed by plants and microbes (Rejmánková et al., 2011; Stone and Plante, 2014). These extracellular enzymes are key agents in organic P mineralization and play important roles in plant response to limited P availability or increasing P demand (Dakora and Phillips, 2002; Burns et al., 2013; Dalling et al., 2016). More recently, phosphatase activity has been determined to be a crucial uncertainty in modeling P cycling in ecosystem models because P mineralization is among the least understood aspects of P dynamics (Reed et al., 2015). Several modeling studies suggest that the magnitude of phosphatase activity could significantly influence CO_2_ uptake in tropical forests (Goll et al., 2012; Yang et al., 2016). Determining the interaction between roots and the bacterial community in regulating phosphatase activity and the factors that influence those interactions is central to understanding P mineralization. However, much of what we currently understand about the role of bacteria in P acquisition is derived from agricultural studies. Although these studies have provided valuable insight, there is a need to extend understanding of those systems to tropical forests, especially with respect to root traits and function (Richardson and Simpson, 2011; Pii et al., 2015). The improved understanding of variations among different tree species of root and bacterial function may provide a means to improve representation of tropical forests in ecosystem models and help us gain a more thorough understanding of how roots and their bacterial community enable tropical forests to thrive in severely limited P soils.

To understand how root and bacterial function differed by tree species and soil P availability, we assessed phosphatase activity in roots and bacterial isolates. Because bacterial community composition may influence this function, we also characterized the bacterial component of the microbial community by sequencing the 16S amplicon. The overarching question was how soil P availability and tree species influenced root and associated bacterial phosphatase activity, as well as root associated bacterial community composition. We hypothesized that (1) phosphatase activity would be higher in areas with low P availability, (2) within a site, tree species would differ in phosphatase activity, and (3) bacterial community composition would primarily depend on tree species. The numerous interactions between roots and the bacterial community determine, in part, P mineralization of tropical ecosystems. Therefore, understanding factors which influence those interactions provides a means to unravel how rhizosphere processes belowground relate to the forest above.

## Materials and Methods

### Study Sites and Tree Species

To examine the influence of P availability on root and bacterial traits, three study sites in the Luquillo mountains of northeastern Puerto Rico (18°30′N, 65°80′W) were chosen to represent a spectrum of P availability. Previous measurements of Rio Icacos (Icacos), El Verde Ridge (Ridge), and El Verde Valley (Valley) indicated lower total P in Icacos and higher total P in Ridge and Valley reflecting differences in parent material. Icacos, formed on quartz-diorite parent material had the lowest total P content (170 ± 25 ppm P) followed by Ridge (290 ± 15 ppm P) and finally, Valley (410 ± 43 ppm P), both of which were on volcaniclastic parent material known to contain higher amounts of soil P (Mage and Porder, 2013). In Icacos, Ridge, and Valley, the most common tree species were sampled for root phosphatase. In Icacos, the tree species sampled were *Cecropia schreberiana* Loefl., *Micropholis garcinifolia* Grisseb, *Cyrilla racemiflora* L., and *Prestoea montana*. In Ridge and Valley, the trees sampled were as follows: *Prestoea montana* Hook, *Dacryodes excelsa* Vahl, and *Manilkara bidentata* Chevalier (Supplementary Table 5).

### Sample Collection

#### Soil Collection and Resin P Assay

At each site, nine cores (2.5 cm diameter) were collected from 0 to 10 cm soil depths to measure site level P availability using the resin P method (Calvert et al., 1993). The resin P method estimates the amount of labile P available for plant and microbial uptake by imitating root removal of phosphate from the soil solution. Charged resin membrane strips in sample soil suspensions attract phosphate ions. Then, the resin strips are washed to remove adhering phosphate ions, resulting in an extract that serves as a proxy for orthophosphate availability in the soil (Van Raij et al., 2009). From each core, 8 g of soil were mixed with 80 mL of distilled water and five resin strips charged with sodium bicarbonate. Samples were shaken for 24 h after which the resin strips were washed of the adsorbed phosphate ions in 50 mL of sulfuric acid. Orthophosphate in the resulting extract was quantified using the Lachat QuikChem 8500. Site level P availability is presented as an average of the nine cores taken at each site.

#### Root Clusters

The first three most distal ends of the root were collected for the root phosphatase assay according to recent studies that indicate functional differences in the root system. The first three distal ends (orders) are associated with resource acquisition in contrast with higher orders which are involved in resource transport (McCormack et al., 2015). Three root clusters (first three orders) from the most common tree species in each site were sampled during November 2015. Root clusters were collected by tracing roots from the base of each individual tree and gently excavating to the terminal three root orders. In total, 12 root clusters were collected from Icacos, 9 root clusters from Ridge, and 9 root clusters from Valley (*n* = 3 per tree species). Collected roots were kept cold during transport using cold packs and shipped overnight to Oak Ridge National Laboratory for further analyses.

#### Bacterial Strain Isolation

To characterize Ridge and Valley bacterial diversity, bacterial isolates and community composition were collected during August 2015. Adhering soil from roots of *D. excelsa* and *P. montana* collected at Ridge and Valley were washed off using 10 mM magnesium sulfate. The resulting slurry was pooled for each tree species at each site before taking three serial dilutions. Each dilution was plated onto solid R2A media (18.2 g Difco^TM^ R2A l^-1^) (Reasoner and Geldreich, 1985). Throughout 1 week, colonies were selected and re-streaked for isolation on fresh R2A solid agar plates. Single colonies of the resulting plates were re-streaked on fresh R2A solid agar plates three times to ensure pure colonies of each isolate. Bacterial stocks of each isolate were made by growing individual colonies overnight in R2A liquid medium at 25°C and mixing the ensuing bacterial culture with an equal volume of 50% glycerol. These solutions were frozen in -80°C and re-streaked onto fresh plates for downstream functional assays.

#### Microbial Community

Culturing bacteria enables insight into bacterial function of members of the bacterial community. In addition, a broader survey of the bacterial community via 16S amplicon sequencing provides the opportunity to understand which phyla are present in the bacterial community, placing into context observed bacterial function. Therefore, rhizosphere soil from Ridge and Valley was collected from *D. excelsa* and *P. montana* roots for bacterial 16S rRNA gene sequencing. Tweezers were used to separate adhering soil from terminal roots. Approximately 50 mg of those roots were washed in 50 ml falcon tubes with milliQ H_2_O before adding rhizosphere soil and pelleting the samples from each tree in Ridge and Valley. A total of 12 samples for DNA extraction from *D. excelsa* and *P. montana* roots were collected (three individuals surveyed per species per site). DNA was extracted from the soil pellet using MoBio PowerSoil kit as per manufacturer’s instructions (Mo Bio Laboratories) and purified using the Zymo clean-up kit (Zymo Research). Samples were then stored in -20°C. Prior to amplification, DNA concentration was normalized to 10 ng μl^-1^.

### Root and Bacterial Function

#### Root Phosphatase

To estimate quantitative phosphorous transformation, PME and PDE activities were measured for the most common tree species at Icacos, Ridge, and Valley. A modified version of the colorimetric soil phosphatase test by Tabatabai and Bremner (1969) was used for root samples with para-nitrophenylphosphate (pNPP) and bis-para-nitrophenylphosphate (bis-pNPP) as a substrate for PME and PDE activities, respectively (Dr. Benjamin L. Turner, personal communication). Enzyme activity for tree species was calculated as the average of all technical replicates for each biological replicate of each species (*n* = 9). Approximately 0.3 ± 0.1 g of roots were weighed with minimal cutting to maintain natural root surface area. Weighed roots were placed between moistened paper towels to prevent drying during the weighing process for other samples. Each weighed root was then placed into a glass vial with 9 mL of 50 mM sodium acetate (pH = 5.0). Following a 5-min equilibration time in a 27°C shaker, 1 mL of 50 mM pNPP was added for the PME assay, or 1 mL of 50 mM bis-pNPP for the PDE assay. Samples were incubated in the shaker for 1 h, and then 0.5 mL of the sample solution was added to 4.5 mL of 0.11 M NaOH to terminate the reaction. Blank solutions were pure 10 mL of 50 mM sodium acetate incubated along with the samples, 0.5 mL of which was added to 4.5 mL of 0.11 M NaOH. Absorbance at 410 nm was read on a Thermo Spectronic 20D and compared to a standard curve generated from para-nitrophenol (pNP), the yellow end-product released from PME or PDE hydrolysis of pNPP.

#### Culture of Bacterial Isolates and Bacterial Phosphomonoesterase Activity

Bacterial PME activity was assessed in isolated strains to estimate potential contribution of bacteria to P mineralization. Each bacterial strain was grown in 1.5 mL R2A overnight before centrifugation at 10,000 rpm for 20 min. A total of 1 mL of the resulting supernatant was added to 1 mL of 50 mM pNPP and 4 mL of modified universal buffer (MUB). MUB consisted of (for 1 L): 12.1 g Tris(hydroxymethyl)aminomethane (THAM or Tris base); 11.6 g maleic acid; 14.0 g citric acid; 6.3 g boric acid; and 1 M sodium hydroxide (pH = 6.5). The solution was then incubated at 27°C for 1 h before terminating the reaction by adding 5 mL of 0.5 M NaOH. Blank solutions consisted of 1 mL of pure R2A culture, 1 mL of 50 mM pNPP, 4 mL of MUB, and 5 mL of 0.5 NaOH. Absorbance at 410 nm was read on a Thermo Spectronic 20D and compared to a standard curve generated from pNP (Satta et al., 1979).

#### Bacterial P Solubilization

Phosphorus (P) solubilization is a key trait in bacteria that is routinely measured to assess bacterial strains for plant growth promoting properties. To induce P solubilization activity of bacterial isolates, each isolate was grown in National Botanical Research Institute’s phosphate growth medium (NBRIP), which requires bacteria to solubilize calcium phosphate to acquire P for growth (Panhwar, 2012). Briefly, NBRIP consisted of (for 1 L): 10 g glucose, 5 g calcium phosphate, 5 g magnesium chloride hexahydrate, 0.25 g magnesium sulfate heptahydrate, 0.2 g potassium chloride, 0.1 g ammonium sulfate, and 15 g agar (Nautiyal, 1999). P solubilization was assessed in 96-well plates via a scaled-down version of the United States Geological Survey (USGS) acid-persulfate digestion method (Patton and Kryskalla, 2003). Samples were plated in triplicate, and the average value of those three wells were used as P solubilization for that isolate. Samples were compared to standard curves between 0.005 and 0.1 mg L^-1^ potassium phosphate and read on a Thermo Spectronic 20D at a wavelength of 880 nm.

### DNA Analysis

#### Preparation for Sequencing

The bacterial V4 16S rRNA region was selectively amplified and barcoded using a two-step PCR approach and using established protocols with PNA blockers to prevent plastid and mitochondrial 16S amplification (Lundberg et al., 2013). Barcoded DNA was sequenced on a single lane of an Illumina MiSeq. Forward primers for the first PCR amplification consisted of three 515F universal primers combined with one 515F Crenarchaeota primer and one 515F TM7 primer. Reverse primers were a mixture of three universal 806R primers (Supplementary Table 1). Thermal cycler conditions for the primary PCRs for soils were 5 cycles of 95°C for 1 min, 50°C for 2 min, and 72°C for 1 min. A total of 30 μL of the primary PCR products were cleaned with 21 μL of Agencourt AMPure beads and eluted in 21 μL of nuclease-free water. For secondary PCRs, the same reverse and forward primers were used in the 50 μL reaction. Thermal cycler conditions for secondary soil PCRs consisted of denaturation at 95°C for 45 s followed by 32 cycles of 94°C for 15 s, annealing at 60°C for 30 s, 72°C for 30 s, and final extension at 72°C for 30 s.

#### Sequence Analysis

Downstream analysis of sequenced bacterial 16S sequences were conducted in MacQIIME (Caporaso et al., 2010). First, bacterial 16S sequences were assigned to respective samples using barcode sequences designated to each sample prior to amplification and sequencing. Sequences were then clustered into OTUs using an open reference OTU picking protocol using UCLUST at a threshold of 97% similarity (Edgar, 2010). Representative sequences were chosen for each OTU and were identified using the Greengenes database. The lowest amount of sequences per sample was 4,363 and the highest was 25,234 with a mean of 12,593. The sampling depth of 7,497 was chosen to include as many samples as possible without compromising diversity analyses with low sampling depths (Supplementary Figures 1, 2). Therefore, samples containing less than 7,497 sequences were removed, which eliminated one *P. montana* sample from Ridge (4,363), one *P. montana* sample from Valley (5,250), and one *D. excelsa* from Valley (6,895). Finally, both mitochondrial and chloroplast sequences were removed. The relative abundances of all the phyla (**Figure 3A**) are based on the percentage of 16S rRNA sequences assigned to each phylum.

### Statistical Analysis

Root phosphatase data were rank transformed prior to performing two-way repeated measures multivariate analysis of variance (MANOVA). Two-way MANOVA avoids errors associated with running multiple ANOVAs because this method can test both enzymes with respect to tree species and site at the same time. The dependent variables were PME and PDE activities, while the independent variables were tree species and site. For each enzyme, three technical replicates of fine root clusters were assayed for three individuals of each tree species at each site. An α = 0.05 was chosen to denote statistical significance of either tree species or site on phosphatase activity. Tukey’s HSD was used to further examine significant differences of phosphatase activity among samples.

To understand how bacterial community composition responded to tree species and site, a weighted UniFrac distance matrix and principal coordinate analysis plot were constructed using the packages ‘phyloseq’ and ‘vegan’ in R (McMurdie and Holmes, 2013; Oksanen et al., 2015). A relative abundance plot was created to show the abundance of phyla in each sample. Principal coordinated analysis (PCoA) was used to visualize the differences between bacterial communities of *P. montana* and *D. excelsa*, where 95% confidence ellipses were drawn around sample points to indicate similarity. Permutational MANOVA (PERMANOVA) was used to statistically test whether bacterial community composition was significantly different between Ridge and Valley and between tree species. An α = 0.05 was also chosen to indicate significance of either site or tree species in determining bacterial community composition.

## Results

### Resin P Availability Is Lowest in Icacos and Highest in Valley

**FIGURE 1:**
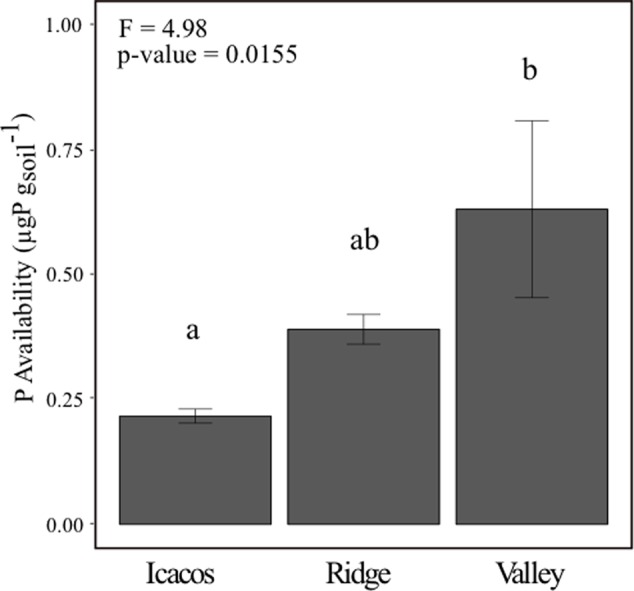
Soil resin P availability across all three sites with letters denoting similarity/dissimilarity of group means. Error bars represent mean ± SE with *n* = 10 (Icacos), *n* = 9 (Ridge), and *n* = 9 (Valley).

Resin P availability increased from Icacos to Ridge to Valley (**Figure 1**) and varied significantly by site *(p*-value < 0.05) (**Table 1**). Post hoc analysis by Tukey’s HSD indicated that Icacos and Valley differed significantly in resin P availability (Supplementary Table 2). Ridge, which was the intermediate site, did not statistically differ from either Icacos or Valley.

**Table 1:** ANOVA of soil resin P across all three sites.

Resin P ANOVA table					
	Degrees of	Sum of	Mean	*F*-value	Pr (>*F*)
	freedom	squares	squares		
Site	2	0.7643	0.3822	4.9789	0.0155^∗^
Residuals	24	1.8421	0.0768		

*^∗^Denotes *p-*value < 0.05.*

### Root Phosphatase Activity Varies with Tree Species and P Availability

**FIGURE 2:**
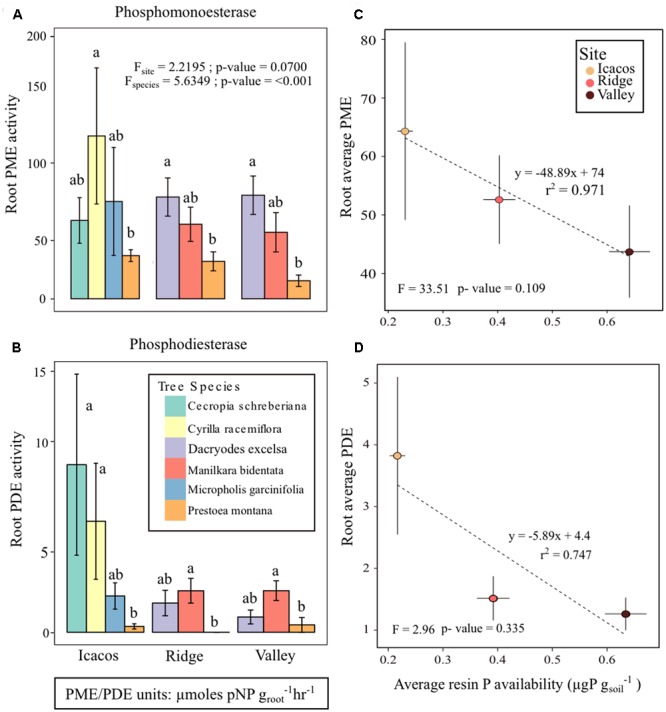
**(A)** Root phosphomonoesterase (PME) activity, **(B)** root phosphodiesterase (PDE) activity, **(C)** average root PME activity plotted with average resin P availability at each site, and **(D)** average root PDE activity with average resin P availability. Letters represent differences in group means among tree species and error bars are mean ± SE with *n* = 3 individual trees per species at each site.

Both PME and PDE were significantly different among tree species (Pillai’s *P* = 0.59; *p-*value < 0.05), though marginally not by site (*p-*value = 0.70) at an α = 0.05 (**Figures 2A,B** and **Table 2**). Furthermore, there was no significant interaction between factors site and tree species. Tukey’s HSD of the two-way repeated measures MANOVA indicated that PME and PDE of *P. montana* differed from all other tree species, except for *M. garcinifolia* (Supplementary Tables 3A,B). The average PME in Icacos was 60.03 ± 15.17 μmol pNP g_root_^-1^hr^-1^, 36.54 ± 7.56 μmol pNP g_root_^-1^hr^-1^ in Ridge, and 24.98 ± 7.88 μmol pNP g_root_^-1^hr^-1^ in Valley. For all tree species, PDE activity was roughly one-tenth of PME. Similar to PME, PDE is highest in Icacos (3.63 ± 6.78 μmol bis-pNP g_root_^-1^hr^-1^), lower in Ridge (1.31 ± 1.76 μmol bis-pNP g_root_^-1^hr^-1^), with the lowest PDE occurring in Valley (1.06 ± 1.37 μmol bis-pNP g_root_^-1^hr^-1^). Differences in tree species means, as denoted by the letters in **Figures 2A,B** for PME and PDE indicated that *P. montana* was significantly different between *C. racemiflora, D. excelsa, and M. bidentata*. In addition, PDE activity of *P. montana* differed from *C. schreberiana (p-*value < 0.05).

**Table 2:** Site and tree species two-way repeated measures MANOVA of root PME and PDE activities.

Site × tree species root phosphatase					
	Degrees of freedom	Pillai	Approximate *F*	Num. degrees of freedom	Den. degrees of freedom	Pr (>*F*)
Site	2	0.1208	2.2195	4	138	0.0700
Species	5	0.5798	5.6349	10	138	<0.001^∗^
Site:Species	2	0.0414	0.7306	4	138	0.5725
Residuals	69					

*^∗^Denotes *p-*value < 0.05.*

The initial MANOVA of root phosphatase as related to site and tree species indicated the significant effect of tree species but not of site in determining enzyme activity. However, a second MANOVA performed by replacing site with resin P availability showed that resin P availability also influenced enzyme activity (Pillai’s *P* = 0.0901; *p*-value = 0.0384) (**Table 3**), though this is due to a change in degrees of freedom. Resin P availability within each site was significant in determining root phosphatase in contrast to site itself which was not. Indeed, average root PME and PDE with respect to average resin P availability exhibited negative correlations (PME *r*^2^ = 0.971; PDE *r*^2^ = 0.747) though neither was significant (PME *p*-value = 0.109; PDE *p*-value = 0.335) (**Figures 2C,D**). In summary, root phosphatase activity is significantly controlled by tree species, not site, though analysis of resin P suggests the importance of soil P availability in co-influencing enzyme activity in the rhizosphere.

**Table 3:** Resin P and tree species two-way repeated measures MANOVA of root PME and PDE.

Resin P × tree species root phosphatase					
	Degrees of freedom	Pillai	Approximate *F*	Num. degrees of freedom	Den. degrees of freedom	Pr (>*F*)
Resin P	2	0.0901	3.4180	2	69	0.0384^∗^
Species	5	0.5946	5.9232	10	140	<0.001^∗^
Resin P:Species	2	0.0357	0.6369	4	140	0.6370
Residuals	69					

*^∗^Denotes *p-*value < 0.05.*

### Bacterial Community Composition in Ridge and Valley Is Influenced by Site

**FIGURE 3:**
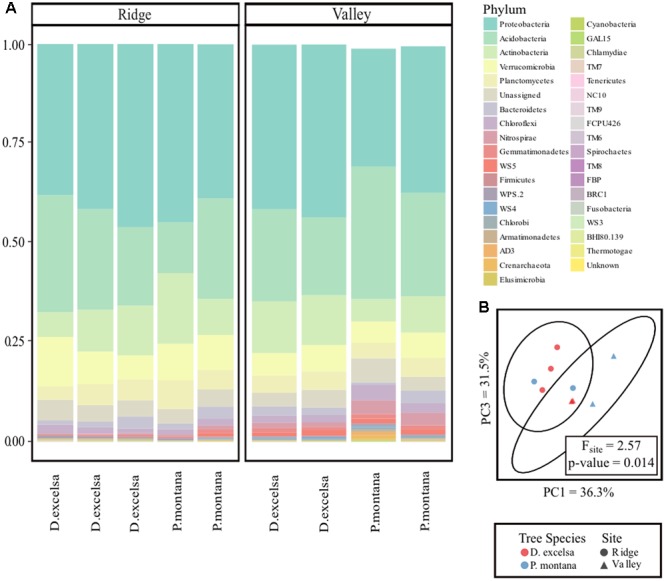
Comparison of bacterial communities between Ridge and Valley sites and *D. excelsa* and *P. montana*. Relative abundance plot of bacterial phyla > 2% of the community **(A)**. One *P. montana* sample in the Valley contained multiple phyla each representing < 2% of the community. These were excluded from the plot resulting in a shorter bar. PCoA with ellipsoids representing 95% confidence intervals **(B)**.

Our analysis of the rhizosphere bacterial community in *P. montana* and *D. excelsa* in Ridge and Valley identified 415 OTUs representing 36 bacterial phyla. The sequence data are stored in NCBI BioProject Accession No. PRJNA412374. Over 50% of the community in both Ridge and Valley came from two phyla: *Proteobacteria* and *Acidobacteria* with no large differences in composition between Ridge and Valley at the phyla level (**Figure 3A**). The proportion of *Proteobacteria* ranged from 29 to 43% in Valley, whereas it was 37 to 45% in Ridge. The proportion of *Acidobacteria* varied more widely from 13 to 45% in Valley and 19 to 34% in the Ridge. The relative abundance plot of bacterial phyla associated with the fine roots of D. *excelsa* and *P. montana* in Ridge and Valley shows no visible differences in relative abundance except for the presence of the *Nitrospirae* phylum found exclusively in Valley.

A weighted UniFrac distance matrix was used to create a PCoA plot with 95% confidence intervals to visually determine similarities among *P. montana* and *D. excelsa* bacterial communities. Points in the PCoA represent each bacterial community, with distances between points indicating similarity or dissimilarity. The resulting plot suggests that bacterial communities are more similar between sites rather than tree species (**Figure 3B**). PERMANOVA using bacterial community composition was used to statistically test whether tree species or site influenced bacterial community composition. At an α = 0.05, PERMANOVA results verify that bacterial community composition is influenced by site, not tree species (*p*-value < 0.05) (**Table 4**).

**Table 4:** PERMANOVA for analysis of similarity/dissimilary among bacterial community from *D. excelsa* and *P. montana*.

Bacterial PME and PDE			
	Degrees of freedom	*F*-value	Pr (>*F*)
Site	1	2.5658	0.014^∗^
Species	1	0.7345	0.642
Permutations	999		

*^∗^Denotes *p-*value < 0.05.*

### Bacterial Isolates from *D. excelsa* Have Higher PME and P Solubilization Activity than Isolates from *P. montana*

**FIGURE 4:**
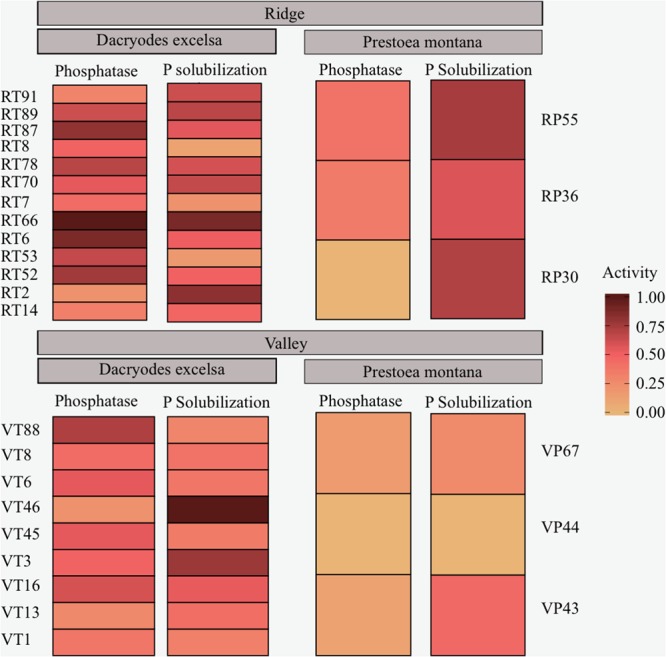
Functional assays of phosphatase activity and P solubilization activity of bacterial isolates from the rhizosphere. Ridge isolates are displayed on top and Valley isolates are on the bottom. Isolates from *D. excelsa* are on the left and isolates from *P. montana* are on the right.

A total of 95 isolates from both Ridge and Valley were tested for PME and P solubilization activity after growing the isolates overnight in a P-deficient media (NBRIP). PME activity was found in 36 isolates with a mean of 0.106 ± 0.021 μmol pNP ml^-1^ in isolates from the rhizosphere of *D. excelsa* and a mean of 0.086 ± 0.005 μmol pNP ml^-1^ from *P. montana.* P solubilization occurred in 28 isolates with higher activity in *D. excelsa* (mean = 6.85 ± 21.58 mg P μL^-1^) relative to *P. montana* (mean = 1.31 ± 1.34 mg P μL^-1^). A majority (67%) of the isolate library was capable of directly enhancing P availability through either PME or P solubilization activity. However, only 32% of the isolate library showed both PME and P solubilization given our assay conditions (**Figure 4**). Of the isolates with both PME and P solubilization, 13 isolates were from Ridge, and nine isolates from Valley. Two-way repeated measures MANOVA conducted on PME and P solubilization activity indicated that tree species was a significant factor in determining both in bacterial isolates (*p*-value < 0.05) but site was not (*p*-value > 0.05) (**Table 5**). Tukey’s HSD indicated a significant difference between *P. montana* and *D. excelsa* in PME (*p-*value = 0.0006), but not P solubilization (*p-*value = 0.66; Supplementary Table 4).

**Table 5:** Two-way repeated measures MANOVA of bacterial PME and P solubilization activity.

Bacterial PME and P solubilization MANOVA					
	Degrees of freedom	Pillai	Approximate *F*	Num. degrees of freedom	Den. degrees of freedom	Pr (>*F*)
Site	1	0.2057	2.9792	2	23	0.0707
Species	1	0.3868	7.2540	2	23	0.0036^∗^
Site:Species	1	0.1416	1.8977	2	23	0.5725
Residuals	24					

*^∗^Denotes *p-*value < 0.05.*

## Discussion

A major challenge of integrating root traits and function is capturing their complex interactions with microbial soil communities. However, it is precisely these interactions between roots and root-associated microbes that direct multiple root functions. Despite their importance, the knowledge of these microbial communities relative to specific root traits is still limited (Bardgett et al., 2014). Roots and root-associated microbes actively release phosphatase to enhance P availability, suggesting that both root and microbial functions must be studied in tandem to fully understand these important mechanisms that contribute to tree growth in P limited tropical forests. Recent simulations indicate that phosphatase activity could be an important factor in regulating the size of the carbon sink given that P acquisition may be a limiting factor in continued growth under changing climates (Yang et al., 2016).

Decades of work have established the importance of the soil environment on determining root and bacterial function (Vitousek and Sanford, 1984; Treseder and Vitousek, 2001; Nannipieri et al., 2011; Spohn et al., 2015), which influenced our choice to study root and bacterial phosphatase activity and bacterial community composition at a collection of sites that increased in P availability from Icacos to Ridge to Valley (Mage and Porder, 2013). Our results showed the same pattern and confirmed the strong influence of site on soil P availability, though the only significant difference in resin P availability was between Icacos and Valley. Our first hypothesis was that phosphatase activity would differ based on soil P availability. Two-way repeated measures MANOVA of root and bacterial phosphatase indicated a significant influence of tree species, though not of site. Replacing site with resin P in a MANOVA, however, did suggest that both tree species and resin P availability influenced phosphatase though this is attributable to a change in the degrees of freedom. Linear regressions of average root PME and root PDE at each site with respect to average resin P availability indicated a negatively correlated trend between root phosphatase and P availability. Lastly, average bacterial PME was higher in isolates from the Ridge than those in the Valley suggesting that site and resin P availability are modulating phosphatase in roots and bacteria. As expected root phosphatase was negatively correlated with resin P availability, supporting the view that root phosphatase may be an important functional trait that forms a critical aspect of nutrient acquisition in low P environments.

Our second hypothesis that phosphatase activity would be influenced by tree species was supported by both root and bacterial two-way repeated measures MANOVAs albeit our limited sample size. Root PME and PDE activities were significantly different between tree species, most notably between *P. montana* samples and four of the five tree species. Similarly, bacterial PME and P solubilization were influenced by host tree species. Post hoc analysis placed *P. montana* samples as a group separate from the other tree species perhaps because *P. montana* was the only monocot. Monocots were shown to differ from dicots in attributes such as more fibrous root systems (Smith and De Smet, 2012), differences in N:P ratios (Han et al., 2004), and differences in mycorrhizal relationships (Cornwell et al., 2001), which may strongly influence functions in the ecosystem. These results are certainly consistent with previous findings that point to the importance of tree species and of plant functional groups in determining phosphatase activity (Hodge, 2004; White and Hammond, 2008; Lambers et al., 2009; Weintraub, 2011; Keller et al., 2013).

The importance of tree species on root phosphatase can also be tied to the combination of conservative and acquisitive traits that regulate tissue P demand and therefore the necessity of acquiring P via root phosphatase. Plants can respond to P limitation by altering functional traits to either increase P efficiency or P acquisition, though it is likely that different tree species use varying combinations of both to adjust to local nutrient supplies (White and Hammond, 2008). Within the tropics, multiple lines of evidence confirm that trees are capable of efficiently using P through high P resorption, P recycling, and reduction of P concentrations in metabolic nucleic acid compounds (Vitousek and Sanford, 1984; Hidaka and Kitayama, 2011). These different mechanisms result in a variety of tissue P requirements for different tree species and therefore different responses to P availability and need for root phosphatase (Ushio et al., 2010; Keller et al., 2013). The lack of an interaction term between site and tree species suggests that tree species is driving differences in root phosphatase activity independently. However, it is important to note that site is likely to be a confounding factor with tree species given that the soil environment can modify these functional traits (Lambers et al., 2006; Richardson et al., 2009; Niu et al., 2013).

Although our alpha level of 0.05 excluded site as a significant determinant of root and bacterial phosphatase activity, we suspect that they are likely influenced by feedbacks between site and tree species. P availability and tree species have consistently been found to regulate root and bacterial function (Treseder and Vitousek, 2001; Costa et al., 2006; Lambers et al., 2006; Haichar et al., 2008; Bardgett et al., 2014; Hinsinger et al., 2015). For example, differences in phosphatase activity between root clusters of the same tree support the notion that heterogeneous distribution of P within each site can cause variation in measured activity. Since roots must respond to local conditions, microsite variations in nutrient content, microbes, and competing roots likely modify phosphatase activity (Baldrian, 2014). Alternatively, differences in phosphatase activity between root clusters of the same individuals and of tree species could be due to variation in the root orders collected and ensuing differences in the microbes attached to root tissue.

Root order was shown to correspond to different roles in the root system where the first and second orders, or the most distal roots, are associated with higher rates of exudation and uptake than those of higher orders typically associated with transport (McCormack et al., 2015). As such, samples with a greater proportion of higher order roots may have shown much less phosphatase activity than samples of the same individual made up of predominantly first- and second-order, absorptive roots. Preliminary observations of *D. excelsa* and *P. montana* roots show that the branching intensity (number of first-order roots per centimeter of second-order roots) of *D. excelsa* (5.58) was higher compared to *P. montana* (2.70) (Daniela Yaffar, personal communication). The difference in branching intensity between *D. excelsa* and *P. montana* may in part explain why phosphatase activity is higher in *D. excelsa* as it has a much higher amount of first-order (resource acquisitive) roots. Similarly, a comparison between lianas and trees demonstrated that roots of lianas seem to favor high P acquisition, correlated with root traits such as greater root branching intensity, higher N and P concentrations, and higher specific root lengths (Collins et al., 2016). Future work will involve examining how root morphology may impact phosphatase activity and whether the release of phosphatase changes depending on the effectiveness of root architectural and morphological traits to find patches of orthophosphate. Indeed, the role of phosphatase activity in determining P mineralization is likely to depend on merging how phosphatase correlates with other root functional traits and the microbial community.

Finally, our third hypothesis that bacterial communities would be largely influenced by tree species was contradicted by results that indicated the significance of site, but not of tree species in determining bacterial community composition. In both Ridge and Valley bacterial communities, the major phyla representing over half of the microbial communities were *Proteobacteria* and *Acidobacteria*, both of which tend to be common in soils (Bulgarelli et al., 2013). However, the phylum *Nitrospirae*, known to be the most diverse group of nitrite-oxidizing bacteria (NOB), only occurred in the Valley (Daims et al., 2015). Conditions in Valley likely favor *Nitrospirae* because of much wetter conditions that create an anoxic soil environment. While there is no question that soil conditions impact the composition of bacterial communities in significant ways through soil pH (Fierer and Jackson, 2006) and microscale heterogeneity (Vos et al., 2013), the intimate association between plants and their root bacterial symbionts certainly affirms the importance of tree species as key factor (Carney and Matson, 2006; Ushio et al., 2008, 2010; Bulgarelli et al., 2013; Philippot et al., 2013). One theory of how rhizosphere microbial communities are formed suggests that soil conditions, such as nutrient availability, initially determine soil microbial community composition. Growing plants then release root exudates which further alter the soil environment, fostering a unique microbial community distinct from bulk soil (Bulgarelli et al., 2013). This two-step model may explain why microbial community composition is more strongly influenced by soil conditions, though the limited sampling size may have obscured stronger effects of tree species on bacterial composition. Interestingly, although bacterial community composition was influenced by site, a two-way repeated measures MANOVA of bacterial PME and P solubilization indicated that these two functions were only influenced by tree species and not site.

PME activity and P solubilization of bacterial isolates were higher in isolates from *D. excelsa* than those from *P. montana*. However, PME was more prevalent in the bacterial isolates collected, despite the importance of P solubilization in enhancing P availability. This might be due to the limitations of the P solubilization assay and the differences in target compounds. First, P solubilization is typically measured using calcium phosphate as a P source, requiring bacterial colonies to solubilize calcium phosphate to survive. However, this favors only those bacterial strains capable of solubilizing calcium phosphate, limiting extrapolation of this function to the broader microbial community. However, P solubilization is a commonly used and relied upon criteria to screen for plant growth promoting activities, thus why we measured it here. Second, phosphatase and P solubilization act on different compounds in the soil. Phosphatase enzymes mineralize organic compounds, which form an abundant portion of total P in tropical forests (Turner and Engelbrecht, 2011). In contrast, P solubilization targets phosphate bound to inorganic compounds, which is a much smaller and more recalcitrant pool (Saghir et al., 2014). It is possible that substrate abundance for PME encourages more members of the bacterial community to express this function. Furthermore, among the 95 isolates tested, only a few isolates were capable of both PME and P solubilization activities, suggesting that it may be uncommon to possess both functions in the larger bacterial community. Given the paradigm that less than 1% of the microbial community can be cultured, our bacterial isolates likely do not capture the full functional potential of the broader microbial community. However, our bacterial isolates provide an opportunity to test bacteria and root interactions in controlled greenhouse settings. Furthermore, our results indicated that tree species played an important role in determining root and bacterial PME activity, supporting efforts to build more species-specific trait understanding of P mineralization.

The root system is highly plastic, responding to variations in P availability through changes in root morphology, architecture, exudation, and interactions with soil microbes (Lambers et al., 2006; Niu et al., 2013). In addition to root and bacterial phosphatase and P solubilization, there are multiple mechanisms critical to understanding how the root system adjusts to P limitation. For example, P deficiency can induce an increase in lateral roots near the soil surface and morphological changes such as increasing root length, root turnover, and higher biomass allocation to roots (Lynch and Brown, 2001). Furthermore, mycorrhizal hyphae are a critical pathway for P uptake because fungal hyphae are much thinner than the smallest roots, enabling a much larger surface area with which to absorb orthophosphate and enhance foraging of soil P sources (Plassard and Dell, 2010; Smith and Smith, 2011). Numerous reviews describe how this intimate association between trees and fungi influence root branching, fine roots, root: shoot ratio, specific root length, and responses to elevated CO_2_ as well as the finer details of the root–fungal interface and the exchange of nutrients (Bucher, 2007; Plassard and Dell, 2010; Smith et al., 2011; Bardgett et al., 2014; Liu et al., 2015). However, our results address the (much less known) bacterial component of the plant microbiome.

Root and bacterial phosphatase activity are central components in plant P acquisition and a source of major uncertainty in understanding P mineralization, forest productivity, and modeling tropical forest growth (Cernusak et al., 2013; Hofhansl et al., 2016). The variation of root phosphatase with tree species is encouraging as it may be an important trait for predicting how different tree species respond in altered climatic regimes. Although tropical forests are immensely diverse, our results with our tree species begin pairing root function with bacterial function and community composition to improve understanding of one critical aspect of P dynamics in tropical forests – phosphatase activity. Our main result was that root and bacterial phosphatase, though not bacterial community composition and P solubilization, vary with tree species and soil P availability. Average values of root phosphatase activity show a promising negative correlation with resin P availability that encourages future empirical and modeling research aimed at building trait-based understanding of P dynamics in tropical forests.

## Conclusion

Production of phosphatase enzymes by both roots and microbes is a crucial function because these enzymes enhance the availability of orthophosphate needed for plant and microbial growth (Marschner et al., 2011). As such, phosphatase activity is an important part of acquiring P. The variation in phosphatase activity among tree species and bacterial communities could be central to understanding how trees adapt to limitations in P supply and how bacterial communities mediate that response. Thus far, roots and microbial communities (if included at all) are modeled as the result of aboveground processes rather than equal determinants of how an ecosystem functions and responds to change (Warren et al., 2015). However, root functional traits and the microbial community are just as central to nutrient cycling, vegetation dynamics, and ecosystem response (Mommer and Weemstra, 2012; Iversen, 2014). Results indicate a negative correlation of root PME activity with increasing P availability, though inference to the broader tropical forest community is limited by the sample size. However, tree species was found to be important in modulating root and bacterial PME activity given local P conditions. These results suggest that efforts to pair root function with microbial community function and composition can indeed provide a platform to unravel complicated rhizosphere interactions that can support the inclusion of roots and microbes in ecosystem models and improve our understanding of key rhizosphere interactions that regulate P mineralization in tropical forests.

## Author Contributions

KC, DW, and RN designed the study. KC, JC, and RN conducted the field studies. KC, CT, AC, T-YL, DP, and DW isolated bacteria from root samples, extracted rhizobacterial DNA, and prepared it for sequencing. KC, CT, AC, and DW analyzed microbial community sequences. KC, CT, and AC conducted root and microbial enzyme analysis. JC performed the P analysis. KC drafted the manuscript. All authors contributed to writing and finalizing the paper, and also read and approved the final manuscript.

## Conflict of Interest Statement

The authors declare that the research was conducted in the absence of any commercial or financial relationships that could be construed as a potential conflict of interest.
