# Supplementary material for: Root and Rhizosphere Bacterial Phosphatase Activity Varies with Tree Species and Soil Phosphorus Availability in Puerto Rico Tropical Forest
Source: Front Plant Sci. 2017 Oct 30;8:1834. doi: 10.3389/fpls.2017.01834 (PMC5670114; doi:10.3389/fpls.2017.01834)
Supplement: Supplementary file 1 [file Data_Sheet_1.DOCX]

**Supplementary Table 1:**

**List of primers used for microbial community PCR amplification**

**Supplementary Figure 1:**

**Rarefaction curves for microbial community sequencing depth 4,363 & 7,497**

**Supplementary Table 2:**

**Resin P Tukey HSD**

| Resin P Tukey multiple comparisons of means | | | | |
| --- | --- | --- | --- | --- |
|  | diff | lwr | upr | p adj |
| Valley-Ridge | 0.2405 | -0.0956 | 0.5767 | 0.1952 |
| Icacos-Ridge | -0.1739 | -0.4918 | 0.1439 | 0.3737 |
| Icacos-Valley | -0.4145 | -0.7427 | -0.0863 | 0.0115* |
| 95% family-wise confidence level | | | | |

***Significant at p- value < 0.05**

**Supplementary Table 3:**

**A Tukey HSD of two-way repeated measures MANOVA of root phosphomonoesterase**

| Root Phosphomonoesterase (PME) Tukey multiple comparisons of means | | | | |
| --- | --- | --- | --- | --- |
|  | diff | lwr | upr | p adj |
| CYRRAC-CECSCH | 0.7545 | -0.5772 | 2.0863 | 0.5626 |
| DACEXC-CECSCH | 0.7284 | -0.3999 | 1.8568 | 0.4162 |
| MANBID-CECSCH | 0.1040 | -1.0243 | 1.2324 | 0.9997 |
| MICGAR-CECSCH | 0.0093 | -1.3726 | 1.3913 | 1.0000 |
| PREMON-CECSCH | -0.6783 | -1.7709 | 0.4142 | 0.4607 |
| DACEXC-CYRRAC | -0.0261 | -1.0923 | 1.0401 | 0.9999 |
| MANBID-CYRRAC | -0.6504 | -1.7167 | 0.4157 | 0.4806 |
| MICGAR-CYRRAC | -0.7452 | -2.0769 | 0.5865 | 0.5759 |
| PREMON-CYRRAC | -1.4329 | -2.4611 | -0.4046 | 0.00156* |
| MANBID-DACEXC | -0.6243 | -1.4222 | 0.1735 | 0.2110 |
| MICGAR-DACEXC | -0.7190 | -1.8475 | 0.4093 | 0.4310 |
| PREMON-DACEXC | -1.4067 | -2.1531 | -0.6604 | 7.60E-06* |
| MICGAR-MANBID | -0.0947 | -1.2231 | 1.0336 | 0.9998 |
| PREMON-MANBID | -0.7824 | -1.5287 | -0.0360 | 0.0344* |
| PREMON-MICGAR | -0.6876 | -1.7802 | 0.4048 | 0.4451 |

**B. Tukey HSD of two-way repeated measures MANOVA of root phosphodiesterase**

| Root Phosphodiesterase (PDE) Tukey multiple comparisons of means | | | | |
| --- | --- | --- | --- | --- |
|  | diff | lwr | upr | p adj |
| CYRRAC-CECSCH | -0.0451 | -1.2586 | 1.1683 | 0.9999 |
| DACEXC-CECSCH | -0.4898 | -1.5180 | 0.5383 | 0.7295 |
| MANBID-CECSCH | -0.1107 | -1.1389 | 0.9174 | 0.9995 |
| MICGAR-CECSCH | -0.5911 | -1.8503 | 0.6681 | 0.7417 |
| PREMON-CECSCH | -1.2310 | -2.2265 | -0.2354 | 0.0069* |
| DACEXC-CYRRAC | -0.4447 | -1.4162 | 0.5268 | 0.7613 |
| MANBID-CYRRAC | -0.0655 | -1.0371 | 0.9059 | 0.9999 |
| MICGAR-CYRRAC | -0.5459 | -1.7594 | 0.6675 | 0.7742 |
| PREMON-CYRRAC | -1.1859 | -2.1228 | -0.2489 | 0.0053* |
| MANBID-DACEXC | 0.3791 | -0.3479 | 1.1061 | 0.6479 |
| MICGAR-DACEXC | -0.1012 | -1.1294 | 0.9269 | 0.9997 |
| PREMON-DACEXC | -0.7411 | -1.4212 | -0.0610 | 0.0247* |
| MICGAR-MANBID | -0.4803 | -1.5085 | 0.5478 | 0.7454 |
| PREMON-MANBID | -1.1203 | -1.8004 | -0.4402 | 0.0001* |
| PREMON-MICGAR | -0.6399 | -1.6354 | 0.3556 | 0.4211 |

**Supplementary Table 4**

**Tukey HSD of two-way repeated measures MANOVA of bacterial PME and P solubilization**

| Bacterial isolates phosphomonoesterase (PME) – Tukey multiple comparison of means | | | | |
| --- | --- | --- | --- | --- |
|  | diff | lwr | upr | p adj |
| PREMON-DACEXC | -1.3794 | -2.1055 | -0.6534 | 0.000619* |

| Bacterial isolates P solubilization – Tukey multiple comparison of means | | | | |
| --- | --- | --- | --- | --- |
|  | diff | lwr | upr | p adj |
| PREMON-DACEXC | -0.1997 | -1.1463 | 0.7470 | 0.6678 |

**Supplementary Figure 2: Principal coordinate analysis comparison of sequencing depths: 4,363 and 7,947**

**Supplementary Table 5: Tree Species**

| Tree Species | Site | Family | Native Range in Puerto Rico | | Climate | Elevation | Tree Height & Diameter |
| --- | --- | --- | --- | --- | --- | --- | --- |
| *Cecropia schreberiana* Loefl. | Icacos | Cecropiaceae | Recently disturbed areas, roadsides and stream banks.  Nearly all forest types, most abundant at mid-elevations | | Subtropical to montane rainforest | 600-900 m | 20 m in height  60 cm in diameter |
| *Micropholis garcinifolia* Grisseb | Icacos | Sapotaceae | Leeward slopes and ridges | | Montane subtropical rain forest | 300 m and higher | 20 m in height  7 m in diameter |
| *Cyrilla racemiflora* L. | Icacos | Cyrillaceae | Ridges and slopes, particularly gaps. | | Subtropical lower montane rain forest | 300-1200 m | 30 m in height  2 m in diameter |
| *Prestoea montana* Hook | Icacos, Ridge, Valley | Arecaceae | | Steep, windward facing slopes, ravines at low elevation, and protected sites at summits | Subtropical moist forest,  subtropical wet forest,  subtropical rain forest,  lower montane wet forest | 400m – summit | 6 - 15 m tall  4 - 20 cm diameter |
| *Dacryodes excelsa* Vahl | Ridge & Valley | Burseraceae | | Northern lower and middle slopes of mountains of Puerto Rico usually on upper slopes and ridges | Subtropical wet forest | 200 – 900 m | 30 m in height  1- 2 m in diameter |

| *Manilkara bidentata* Chevalier | Ridge & Valley | Sapotaceae | Moist coastal and limestone forests & lower mountain forests | Subtropical moist, subtropical wet, subtropical rain forest | Up to 60m | 30 m in height  1 m in diameter |
| --- | --- | --- | --- | --- | --- | --- |

Tree species table references

1. Brokaw, Nicholas V. L. “Cecropia schreberiana in the Luquillo Mountains of Puerto Rico.” The Botanical Review, vol. 64, no. 2, 1998, pp. 91–120., doi:10.1007/bf02856580.
2. Ewel, J. J., & Whitmore, J. L. (1973). The ecological life zones of Puerto Rico and the U.S.Virgin Islands. Forest Service Research, 72. https://doi.org/10.1017/CBO9781107415324.004
3. Little, E. L., & Wadsworth, F. H. (1964). Common trees of Puerto Rico and the Virgin Islands. Agricultural Handbook No. 249 . U.S. Department of Agriculture, Washington, DC, (249), 416–417. https://doi.org/10.5962/bhl.title.4135
4. Lugo, Ariel E. and Wadsworth, Frank H. “Dacryodes excelsa Vahl.” Northeastern Area State & Private Forestry, USDA Forest Service, www.na.fs.fed.us/spfo/pubs/silvics_manual/volume_2/dacryodes/excelsa.htm. Accessed 15 Sept. 2017.
5. Weaver, P. L. (2010). Forest structure and composition in the lower montane rain forest of the Luquillo mountains, Puerto Rico. Interciencia, 35(9), 640–646.
6. Weaver, P. L. “Ausubo, Balata.” Northeastern Area State & Private Forestry, USDA Forest Service, www.na.fs.fed.us/spfo/pubs/silvics_manual/volume_2/manikara/bidentata.htm. Accessed 15 Sept. 2017.
